# Roflumilast attenuates allergen-induced inflammation in mild asthmatic subjects

**DOI:** 10.1186/1465-9921-12-140

**Published:** 2011-10-26

**Authors:** Gail M Gauvreau, Louis-Philippe Boulet, Christine Schmid-Wirlitsch, Johanne Côté, MyLinh Duong, Kieran J Killian, Joanne Milot, Francine Deschesnes, Tara Strinich, Richard M Watson, Dirk Bredenbröker, Paul M O'Byrne

**Affiliations:** 1 Department of Medicine, McMaster University, Hamilton, Ontario, Canada; 2 Institut Universitaire de Cardiologie et de Pneumologie de Québec, Quebec City, Quebec, Canada; 3 NYCOMED GmbH, Konstanz, Germany

**Keywords:** Allergic asthma, allergen challenge, PDE4 inhibitor, inflammation, sputum, neutrophils, eosinophils

## Abstract

**Background:**

Phosphodiesterase 4 (PDE4) inhibitors increase intracellular cyclic adenosine monophosphate (cAMP), leading to regulation of inflammatory cell functions. Roflumilast is a potent and targeted PDE4 inhibitor. The objective of this study was to evaluate the effects of roflumilast on bronchoconstriction, airway hyperresponsiveness (AHR), and airway inflammation in mild asthmatic patients undergoing allergen inhalation challenge.

**Methods:**

25 subjects with mild allergic asthma were randomized to oral roflumilast 500 mcg or placebo, once daily for 14 days in a double-blind, placebo-controlled, crossover study. Allergen challenge was performed on Day 14, and FEV_1 _was measured until 7 h post challenge. Methacholine challenge was performed on Days 1 (pre-dose), 13 (24 h pre-allergen), and 15 (24 h post-allergen), and sputum induction was performed on Days 1, 13, 14 (7 h post-allergen), and 15.

**Results:**

Roflumilast inhibited the allergen-induced late phase response compared to placebo; maximum % fall in FEV_1 _(p = 0.02) and the area under the curve (p = 0.01). Roflumilast had a more impressive effect inhibiting allergen-induced sputum eosinophils, neutrophils, and eosinophil cationic protein (ECP) at 7 h post-allergen (all p = 0.02), and sputum neutrophils (p = 0.04), ECP (p = 0.02), neutrophil elastase (p = 0.0001) and AHR (p = 0.004) at 24 h post-allergen.

**Conclusions:**

This study demonstrates a protective effect of roflumilast on allergen-induced airway inflammation. The observed attenuation of sputum eosinophils and neutrophils demonstrates the anti-inflammatory properties of PDE4 inhibition and supports the roles of both cell types in the development of late phase bronchoconstriction and AHR.

**Trial Registration:**

ClinicalTrials.gov: NCT01365533

## Background

Asthma is characterized by the presence of cough, wheeze, dyspnea, reversible airway obstruction and airway hyperresponsiveness. Eosinophils are cells recognized to be a key feature of allergic asthma [1], however patients with severe asthma have increases in both eosinophils and neutrophils in their sputum [2]. Furthermore, severe asthma exacerbations are associated with bronchial mucosal eosinophilia and neutrophilia, as well as upregulation of CXC chemoattractants and their receptors [3]. Although current asthma therapies such as corticosteroids are effective in inhibiting eosinophilic inflammation through Th2 suppression, they may enhance neutrophil accumulation into the airways and until now therapies that effectively suppress neutrophilic inflammation have been lacking [4,5].

The intracellular signalling molecules, cyclic adenosine monophosphate (cAMP) and cyclic guanosine monophosphate (cGMP), are implicated in the pathophysiology of asthma; they promote smooth muscle relaxation and inhibit inflammation [6]. A novel approach for therapeutic intervention in asthma is through regulation of the phosphodiesterase (PDE) activity, which is the only cellular pathway available for degradation of cAMP and cGMP [7]. Roflumilast has been shown to improve lung function and reduce exacerbations in chronic obstructive pulmonary disease (COPD) [8,9] and has recently been approved in the EU and Canada for oral once-daily treatment of severe COPD.

Roflumilast is a selective inhibitor of the PDE4 isoform which is specific for cAMP degradation, and is expressed in several effector cells central to the pathophysiology of asthma including eosinophils, neutrophils and lymphocytes [10,11]. As a significant increase in PDE4 activity has been reported in patients with asthma or allergy compared with healthy individuals [12,13], it is therefore of interest to explore PDE4 inhibition as a potential therapeutic option for treatment of atopic asthma, given the proposed anti-inflammatory mode of action [14].

Subjects with allergic asthma develop an immediate IgE-mediated early asthmatic response (EAR) following inhalation of a sufficient dose of an allergen to which they are sensitized [15]. Up to 50% of these subjects also develop a late asthmatic response (LAR) beginning 3 to 4 hours after allergen inhalation challenge [16], and an associated elevation in levels of eosinophils, basophils and mast cells, other effector cells including T lymphocytes [17-20], and Th2-related cytokines and chemokines [18,20-22]. Roflumilast is a potent and targeted PDE4 inhibitor, approved by the European Commission as an add-on to bronchodilator therapy for the treatment of severe chronic obstructive pulmonary disease (COPD) associated with chronic bronchitis in adults with a history of frequent exacerbations, and targets the underlying inflammation in COPD. We hypothesized that roflumilast would attenuate allergen-induced LAR and airway hyperresponsiveness (AHR) through inhibition of airway inflammation. Some of the results of this study have been previously reported in the form of an abstract [23].

## Methods

### Subjects

Forty-seven non-smoking subjects with mild atopic stable asthma underwent screening for this study. Subjects were required to have a forced expiratory volume in one second (FEV_1 _) ≥ 70% of predicted and baseline methacholine PC_20 _(the provocative concentration of methacholine causing a 20% fall in FEV_1_) ≤ 16 mg/mL. Subjects had no other lung disease, no lower respiratory tract infection or worsening of asthma for 6 weeks before screening, and avoided exposure to sensitizing allergens apart from house dust mite. Subjects were steroid-naïve and used infrequent inhaled beta_2_-agonist for treatment of asthma. Beta_2_-agonist and caffeinated beverages were withheld for at least 8 h before laboratory visits. Twenty-five subjects, 10 male/15 female, aged 18-54 years old (Table 1) met all criteria including the development of allergen-induced EAR and LAR. EAR was defined by an acute fall in FEV_1 _≥ 20% within 2 h following allergen challenge, and LAR was defined by a fall in FEV_1 _≥ 15% between 3 h and 7 h following allergen challenge.

**Table 1:** Mean subject demographics and baseline characteristics (range)

Parameter	Intent to treat population N = 25
Age, years	28.7 (18 - 54)

Gender, males/females	10/15

History of asthma, years	15.3 (0 - 43)

FEV_1 _% predicted	92.8 (75.9 - 117.1)

Methacholine PC_20_, mg/ml	4.0 (0.3 - 14.4)

Smokers	none

Legend: FEV_1_: forced expiratory volume in 1 second; PC_20_: provocative concentration causing a 20% fall in FEV_1_.

### Study Design

**Figure 1:**
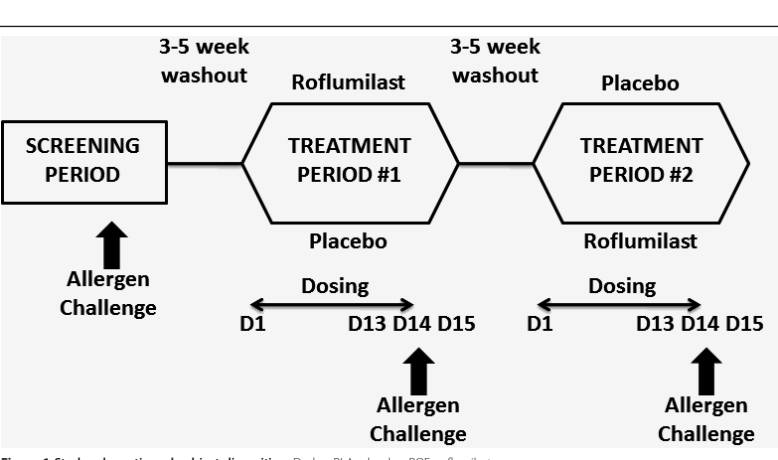
**Study schematic and subject disposition**. D: day; PLA: placebo; ROF: roflumilast.

This trial was a two-center, double-blind, randomized, placebo-controlled, cross-over study, comparing 14 days treatment with roflumilast, 500 mcg, with placebo (Figure 1). The primary outcome was the effect of roflumilast on allergen-induced airway eosinophilia. Secondary outcomes were the allergen-induced EAR, LAR, AHR, steady-state pharmacokinetics (PK) of roflumilast and roflumilast N-oxide, safety and tolerability of roflumilast, and allergen-induced airway metachromatic cells (MCC) and airway inflammatory cell mediator levels. The study was registered with http://clinicaltrials.govNCT01365533.

The study was carried out from December 2004 to July 2005. Each site was given a block of kits containing drug and placebo. The randomization code was computer-generated, and eligible subjects were assigned the next available kit at the site by study staff. All subjects and trial staff remained blinded to the random order during the trial.

The study was approved by the ethics research board of the respective institutions, and signed informed consent of all subjects was obtained. Screening of subjects was performed over 1 week and included 3 consecutive days for assessment of responses to inhaled allergen challenge (Figure 1). Those who developed a fall in FEV_1 _of ≥ 20% within 2 h post allergen (EAR), and ≥ 15% between 3 and 7 h post allergen (LAR) were randomized to two treatment periods separated by 3-5 weeks. To enter a treatment period, methacholine PC_20 _was required to be within one doubling dose of that measured during screening, and FEV_1 _≥ 70% of predicted. Methacholine challenge and sputum induction were performed in the morning of Day 1 (before dosing), Day 13 (24 h pre allergen challenge), and Day 15 (24 h post allergen challenge). Allergen challenge was performed on the morning of Day 14, and sputum was induced 7 h post allergen challenge. Blood was collected for pharmacokinetics throughout Day 14.

### Laboratory Procedures

#### Study Medication

Patients received one roflumilast 500 mcg or placebo tablet orally each morning after breakfast (taken with at least 200 mL of fluid) for 14 consecutive days. The first dose of study drug was administered in the laboratory after all study procedures were completed. Subjects self-administered the study drug from Day 2 to Day 12. Doses on Days 13 and 14 were administered in the laboratory approximately 15 minutes before study procedures. Roflumilast 500 mcg was selected because it is well tolerated and effective in asthma control at this dose [24].

#### Methacholine Inhalation Challenge

Methacholine PC_20 _was measured as described by Cockcroft [25] using tidal breathing from a Wright nebulizer. The test was terminated when a fall in FEV_1 _of at least 20% of the baseline value occurred.

#### Allergen Inhalation Challenge

FEV_1 _was required to be within 10% of baseline to proceed with the challenge. Allergen inhalation was performed as described by O'Byrne and colleagues [16] using the concentration of allergen extract determined from a formula described by Cockcroft and coworkers [26]. During the screening period, doubling concentrations of allergen were administered until a ≥ 20% fall in FEV_1 _at 10 minutes post allergen was reached. FEV_1 _was then measured at regular intervals until 7 h after allergen inhalation. Only subjects developing a LAR of ≥ 15% fall in FEV_1 _during the screening period were eligible to participate in the study. All subjects inhaled the same dose of allergen for each of the two treatment periods. EAR was defined as the largest fall in FEV_1 _within 2 h after allergen challenge, and LAR was defined as the largest fall in FEV_1 _between 3 h and 7 h after allergen challenge. The early and late responses area under the curve (AUC) were calculated from 0-2 h and 3-7 h, respectively (AUC 0-2h, AUC 3-7h) by plotting the response using graphics software that calculated the area of the FEV_1_-time response and expressed as liters by hours (Lxh).

#### Sputum Analysis

Sputum was induced and processed using the method described by Pizzichini and co-workers [27]. The total cell count was determined using a Neubauer hemocytometer chamber (Hausser Scientific, Blue Bell, PA) and expressed as the number of cells per gram of sputum. Cells were prepared on 4 glass slides. Two slides were stained with Diff Quik (American Scientific Products, McGaw Park, IL) and duplicate differential counts (400 cells/slide) were averaged. Two slides were fixed in Carnoy's solution (60% ethanol, 30% chloroform and 10% glacial acetic acid), stained with toluidine blue (Sigma-Aldrich, St. Louis,. MO) which stains the nuclei of all cells pale blue, and glycosaminoglycan-containing granules in the cytoplasm of mast cells and basophils stain metachromatically red/purple. Five thousand cells on each of the two slides were assessed for positive metachromatic staining, and expressed as the number of metachromatic cells per gram of sputum. Supernatant was stored at -70°C and assayed in duplicate using commercial ELISAs for interleukin (IL)-8, myeloperoxidase and neutrophil elastase (R&D Systems, Minneapolis, MN). Eosinophil cationic protein was measured using UniCap (Somagen Diagnostics, Edmonton, AB).

#### Pharmacokinetic Assessments

Blood was collected from 15 subjects at one trial site for pharmacokinetic assessments on Day 14 of each treatment period. Blood was collected pre-dose and post-dose at 15 min, 30 min, 60 min, 90 min, 2 h, 3 h, 4 h, 6 h, 8 h, 12 h, and 24 h. Venous blood was withdrawn into vacuum blood collection tubes containing lithium heparin, and plasma was separated within 30 min by centrifugation at 1000 g for 10 min in a refrigerated centrifuge. Plasma samples were frozen at -20°C then assayed for roflumilast and roflumilast-N-oxide. Analytical measurements of roflumilast and roflumilast N-oxide were performed by a validated method using high performance liquid chromatography (HPLC) coupled with tandem mass spectrometry (HPLC-MS/MS) and allowed an LLOQ of 0.1 μg/L for both, roflumilast, and roflumilast N-oxide using 0.4 mL human plasma.

#### Statistical Analysis

The per-protocol data were analyzed to accommodate the crossover design, thereby removing data from subjects not completing both study periods. Data are presented from 22 subjects as mean ± standard error of the mean (SEM) unless otherwise noted. Methacholine PC_20_, sputum cell numbers and mediator levels were log-transformed prior to analyses. An ANCOVA adopted for the crossover design was performed to evaluate the effects of treatment and period; fixed factors were age, gender, value at the end of washout, and center. The random factor patient nested in sequence was included in the model. It was assumed that the random errors were independent and identically distributed with mean zero and equal variance.

## Results

Two subjects withdrew prematurely from the study due to adverse events (depression, asthma), and one subject was excluded for using inhaled corticosteroids during the trial. Statistical analyses were carried out on the 22 subjects who completed the study per protocol. Baseline characteristics are shown in Table 1.

### Sputum Inflammatory Cells

**Figure 2:**
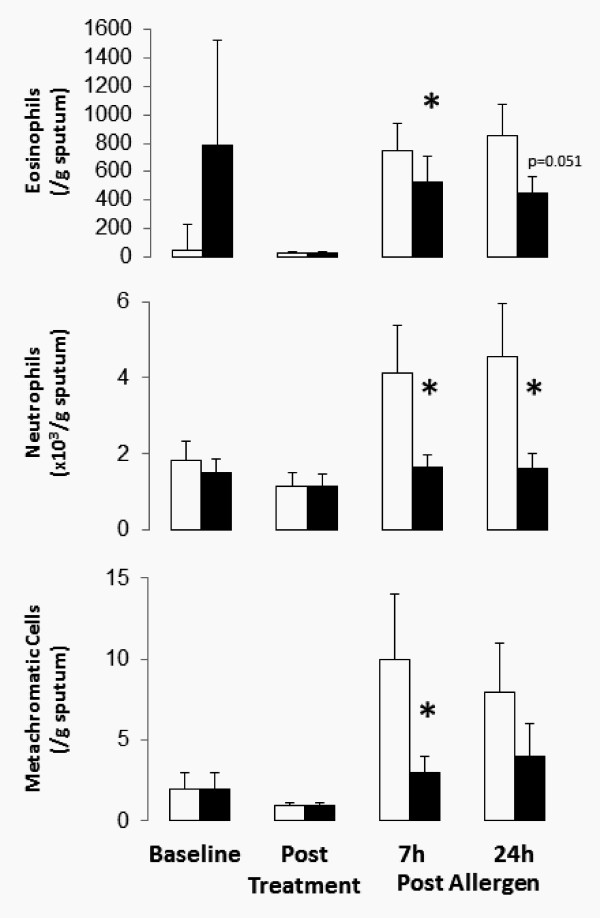
**Mean sputum inflammatory cells (± SEM) at baseline before treatment with roflumilast (solid bars) or placebo (open bars), after 13 days' treatment, and 7 h and 24 h after allergen challenge**. Roflumilast significantly inhibited the allergen-induced influx of sputum eosinophils, neutrophils and metachromatic cells (p < 0.05).

Roflumilast treatment had a significant effect on the total leukocyte count in sputum, thus cell populations are shown as absolute numbers rather than percentages. After allergen inhalation, with placebo treatment, sputum eosinophils increased from a pre-allergen baseline of 28 ± 6/g to 744 ± 199/g at 7 h and 850 ± 227/g at 24 h post-allergen (both p < 0.0001). Compared with placebo, roflumilast inhibited the allergen-induced increase in sputum eosinophils from a pre-allergen value of 29 ± 12/g to 530 ± 182/g at 7 h (p = 0.015) and 446 ± 120/g (p = 0.051) at 24 h post-allergen (Figure 2).

With placebo treatment, after allergen inhalation, sputum neutrophils increased from a pre-allergen baseline of 1.15 ± 0.34 × 10^3^/g to 4.13 ± 0.1.26 × 10^3^/g and 4.56 ± 0.1.39 × 10^3^/g at 7 and 24 h post-allergen (both p < 0.0001). Compared with placebo, roflumilast inhibited the allergen-induced increase in sputum neutrophils from 1.13 ± 0.33 × 10^3^/g pre-allergen to 1.66 ± 0.32 × 10^3^/g at 7 h and 1.61 ± 0.38 × 10^3^/g at 24 h post-allergen (p = 0.017 and p = 0.04, respectively) (Figure 2).

With placebo, allergen inhalation increased sputum MCC (basophils and mast cells) from a pre-allergen baseline of 1.0 ± 1.0/g before challenge, to 10 ± 4.0/g (p < 0.0001) at 7 h, and 8.0 ± 3.0/g (p = 0.002) at 24 h post-allergen. Compared with placebo, roflumilast inhibited the allergen-induced increase in sputum MCC from pre-allergen value of 1.0 ± 1.0/g before to 3.0 ± 1.0/g and 4.0 ± 2.0/g at 7 and 24 h post-allergen (p = 0.003 and 0.37, respectively) (Figure 2).

After 13 days of dosing, subjects receiving roflumilast had no change in baseline numbers of sputum eosinophils, neutrophils, or MCC (basophils and mast cells) (Figure 2).

#### Sputum Fluid Phase Mediators

**Figure 3:**
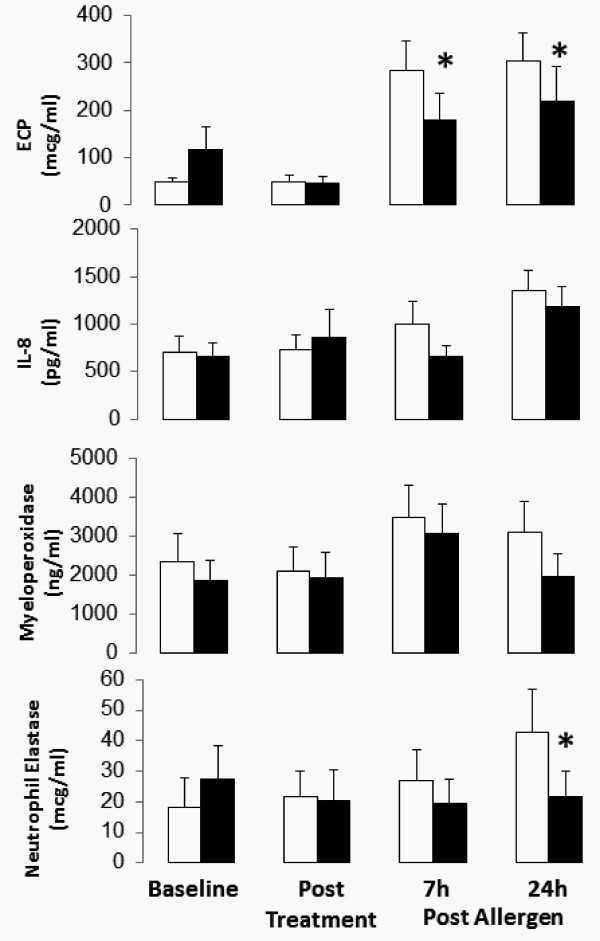
**Mean sputum fluid phase mediators (± SEM) at baseline before treatment with roflumilast (solid bars) or placebo (open bars), after 13 days' treatment, and 7 h and 24 h after allergen challenge**. Roflumilast significantly inhibited the allergen-induced increased level of eosinophil cationic protein (ECP) and neutrophil elastase (NE) (p < 0.05).

The sputum supernatant was assayed for mediators associated with the activation and degranulation of eosinophils and neutrophils. With placebo treatment, the pre-allergen level of ECP rose significantly from 50.4 ± 13.8 mcg/mL to 283.2 ± 62.0 mcg/mL and 304.5 ± 58.5 mcg/mL at 7 h and 24 h post-allergen (p < 0.001). After roflumilast, this increase was attenuated from pre-allergen value of 45.5 ± 15.0 mcg/mL to 178.2 ± 57.6 mcg/mL and 219.9 ± 72.5 mcg/mL at 7 h and 24 h post-allergen, respectively (both p < 0.02). The moderate increase in neutrophil elastase from 21.9 ± 10.2 mcg/mL pre-allergen to 43.0 ± 13.9 mcg/mL at 24 h post-allergen following placebo treatment was significantly attenuated by roflumilast, from 20.6 ± 9.8 mcg/mL pre-allergen to 21.5 ± 8.6 mcg/mL at 24 h post-allergen (p = 0.0001). Post-allergen levels of myeloperoxidase and IL-8 were consistently lower with roflumilast treatment, however not statistically different than placebo (p > 0.05) (Figure 3).

After 13 days of dosing, subjects receiving roflumilast had no change in the level of ECP, IL-8, myeloperoxidate or neutrophil elastase (Figure 3).

#### Allergen-Induced Airway Responses

**Figure 4:**
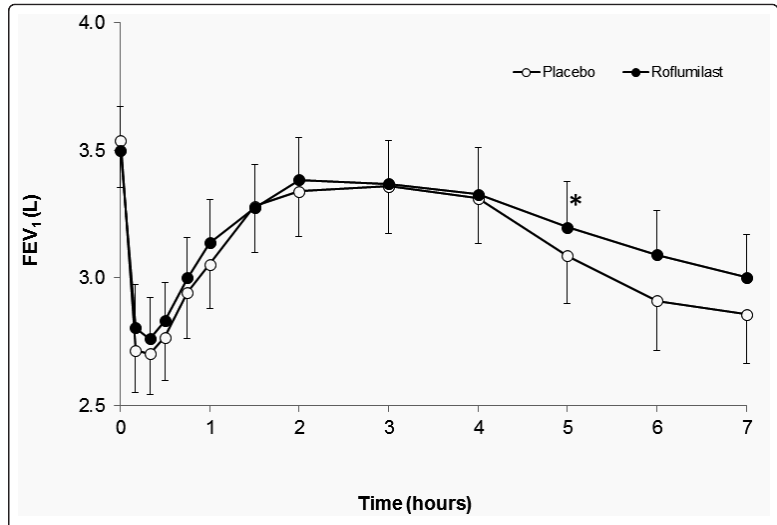
**Mean percent fall in FEV_1 _(± SEM) during the early and late asthmatic responses**. Roflumilast significantly inhibited the area of the early and late responses (p < 0.05). FEV1: forced expiratory volume in 1 second.

There was no difference in the baseline FEV_1 _after 14 days of treatment with roflumilast or placebo (3.50 ± 0.18 L vs 3.54 ± 0.17 L, respectively, p = 0.28. Both allergen-induced early and late falls in FEV_1 _were significantly inhibited by roflumilast (early AUC 0-2 h -0.78 ± 0.10 Lxh compared to placebo -0.94 ± 0.12 Lxh, (p = 0.047) and late AUC 3-7 h -1.19 ± 0.25 Lxh compared to placebo -1.73 ± 0.29 Lxh, (p = 0.013). The maximum change in FEV_1 _during the early response was -0.87 ± 0.08 L (23.4% fall from baseline) with roflumilast compared to -0.93 ± 0.10 L (26.2% fall from baseline) with placebo (p = 0.12). The maximum change in FEV_1 _during the late response was -0.57 ± 0.09 L (16.2% fall from baseline) with roflumilast compared to -0.73 ± 0.10 L (20.7% fall from baseline) with placebo (p = 0.02) (Figure 4).

#### Airway Hyperresponsiveness

The methacholine PC_20 _was unchanged following 13 days' treatment with placebo or roflumilast (p > 0.05), and fell significantly 24 h post-allergen with both placebo and roflumilast (p = 0.022 and < 0.0001, respectively). However, roflumilast significantly attenuated the allergen-induced fall in AHR from 5.64 ± 1.26 mg/mL pre-allergen to 3.65 ± 0.81 mg/mL 24 h post-allergen, compared to placebo from 5.39 ± 1.18 mg/mL pre-allergen to 1.80 ± 0.42 mg/mL 24 h post-allergen (p = 0.004).

#### Safety

One subject under treatment with roflumilast experienced a serious adverse event of depression and was withdrawn from the study. This subject did not have a history of depression. Headache was the most frequently reported adverse event (AE) with roflumilast treatment (4 roflumilast versus 0 placebo), followed by nausea (2 roflumilast versus 0 placebo). The majority of AEs were not considered related to study medication. There were no significant differences between treatments in median values of hematology and blood chemistry variables.

#### Pharmacokinetics in Plasma

**Figure 5:**
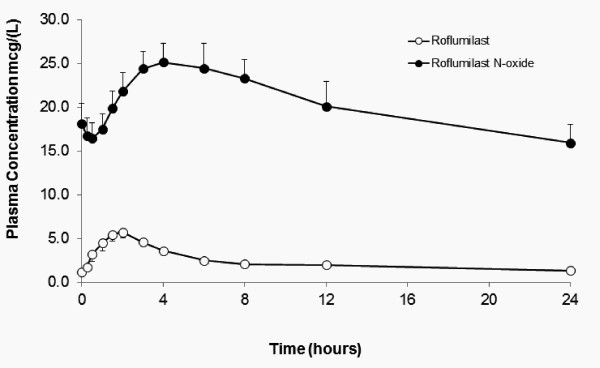
**Mean plasma concentration (± SEM) of roflumilast and the active metabolite roflumilast N-oxide at Day 14**.

Pharmacokinetic profiles were obtained from 15 subjects at Day 14. Following repeated oral doses of roflumilast, estimates of roflumilast and the active metabolite N-oxide roflumilast at Day 14 show maximum plasma concentrations of 7.04 ± 0.65 mcg/L and 26.8 ± 2.59 mcg/L reached at 1.83 ± 0.27 h and 4.37 ± 0.5 h, respectively (Figure 5). The pharmacokinetic profiles and estimates of roflumilast and roflumilast N-oxide are similar to those in previous pharmacokinetic studies in healthy volunteers [28].

## Discussion

The orally active PDE4 inhibitors roflumilast and CDP840, given either as a single dose or regularly are known to inhibit the allergen-induced LAR and AHR [29-31], and rolfumilast has been shown to reduce sputum eosinophil and neutrophils in subjects with COPD [32]. We hypothesized that oral roflumilast would also attenuate allergen-induced airway inflammation, including neutrophilia, which is not generally a target for asthma treatment asthma [3]. This study examined the safety of 14 days' oral treatment with roflumilast 500 mcg in patients with mild asthma and its effects on allergen-induced airway inflammation. The results show that roflumilast attenuated allergen-induced accumulation of inflammatory cells and inflammatory mediators.

In asthma, there is an increase in the number of inflammatory cells within the airways. It is believed that through the release of their mediators and enzymes [4-6] these effector cells contribute to the persistence of inflammation, AHR and increased bronchial tone [1-3]. Roflumilast is a selective PDE4 inhibitor, this inhibition leads to increased cAMP levels in inflammatory cells, rendering them less likely to respond to stimuli. Thus, cell functions, including chemotaxis, survival and activation, which are mediated through cAMP, will be impaired with roflumilast treatment. Roflumilast has been shown to block inflammatory cell influx through inhibition of P- and E-selectin expression on endothelial cells, and CD11b expression on neutrophils [33], and blocks the release of various inflammatory mediators including TNF-alpha, LTB_4_, IL-4 and IL-5 [34]. In keeping with the proposed broad anti-inflammatory effects of roflumilast, the allergen-induced increase in eosinophils, neutrophils, MCC and total leukocyte counts in sputum were inhibited with active treatment. This inhibitory effect on inflammatory cell numbers was also observed in the sputum of subjects with COPD where 500 mcg roflumilast once daily for 4 weeks decreased the total cell count [32].

We hypothesized that roflumilast would suppress the migration and activation of cells involved in the allergic immune response in the airways following allergen inhalation challenge, and thus reduce the physiological responses of EAR, LAR and AHR. This study demonstrates that roflumilast attenuates allergen-induced physiologic responses in subjects with mild allergic asthma, albeit the effect is small as compared to inhaled corticosteroids [35]. Inhibition of physiologic responses is coincident with a larger inhibition of airway inflammation, especially the neutrophil population. Airway neutrophilia has been shown to be elevated in severe asthma, however this could be due, in part, to inhaled corticosteroid treatment [36]. As such, the role of the neutrophil in asthma is still uncertain. Mast cells and basophils are positioned to play a major role in the development of the EAR following IgE-dependent release of histamine and cysteinyl leukotrienes. Inhibition of activation of MCC in sputum by inhaled allergen provides one mechanism for inhibition of the EAR by roflumilast. Likewise, reduced accumulation of eosinophils, neutrophils and their mediators in the airways post allergen reduces inflammatory signals that contribute to development of the LAR. Inhibition of allergen-induced EAR and LAR and AHR is unlikely to be mediated through smooth muscle relaxation because we did not observe improvements in FEV_1 _post-dosing consistent with the lack of direct bronchodilatory activity [37].

## Conclusions

This is the first study to demonstrate inhibition of both allergen-induced airway eosinophilia and neutrophilia with pharmacological intervention, and suggests that the broad anti-inflammatory properties of roflumilast may be effective in attenuating the airway neutrophilic inflammation, which is not well-controlled by corticosteroids.

## List of abbreviations

AE: adverse event; AHR: airway hyperresponsiveness; AUC: area under the curve; cAMP: cyclic adenosine monophosphate; cGMP: cyclic guanosine monophosphate; COPD: chronic obstructive pulmonary disease; EAR: early asthmatic response; ECP: eosinophil cationic protein; FEV_1 _: forced expiratory volume in one second; IL: interleukin; LAR: late asthmatic response; MCC: metachromatic cells; PC_20 _: The provocative concentration of methacholine causing a 20% fall in FEV_1_; PDE4: phosphodiesterase 4; PK: pharmacokinetics; SEM: standard error of the mean.

## Competing interests

This study was funded by NYCOMED. CSW and DB are employees of NYCOMED. The remaining authors have no conflict of interest to declare.

## Authors' contributions

GMG and PO'B designed the study and together with CS, DB and L-PB wrote the manuscript. JC, KJK and MD were responsible for medical procedures. RW, FD, JM, and TS performed study analyses. All authors read and approved the final manuscript.

## References

[B1] Fahy JV Wong H Liu J Boushey HA Comparison of samples collected by sputum induction and bronchoscopy from asthmatic and healthy subjects Am J Respir Crit Care Med 1995 152 53 8 7599862 10.1164/ajrccm.152.1.7599862

[B2] Shannon J Ernst P Yamauchi Y Olivenstein R Lemiere C Foley S Cicora L Ludwig M Hamid Q Martin JG Differences in airway cytokine profile in severe asthma compared to moderate asthma Chest 2008 133 420 426 10.1378/chest.07-1881 18071017

[B3] Qiu Y Zhu J Bandi V Guntupalli KK Jeffery PK Bronchial mucosal inflammation and upregulation of CXC chemoattractants and receptors in severe exacerbations of asthma Thorax 2007 62 475 482 10.1136/thx.2006.066670 17234659 PMC2117215

[B4] Keatings VM Jatakanon A Worsdell YM Barnes PJ Effects of inhaled and oral glucocorticoids on inflammatory indices in asthma and COPD Am J Respir Crit Care Med 1997 155 542 548 9032192 10.1164/ajrccm.155.2.9032192

[B5] Nguyen LT Lim S Oates T Chung KF Increase in airway neutrophils after oral but not inhaled corticosteroid therapy in mild asthma Respir Med 2005 99 200 207 10.1016/j.rmed.2004.06.007 15715187

[B6] Schudt C Gantner F Tenors H Hatzelmann A Therapeutic potential of selective PDE inhibitors in asthma Pulm Pharmacol Ther 1999 12 123 129 10.1006/pupt.1999.0182 10373395

[B7] Beavo JA Cyclic nucleotide phosphodiesterases: functional implications of multiple isoforms Physiol Rev 1995 75 725 748 7480160 10.1152/physrev.1995.75.4.725

[B8] Calverley PM Rabe KF Goehring UM Kristiansen S Fabbri LM Martinez FJ M2-124 and M2-125 study groups Roflumilast in symptomatic chronic obstructive pulmonary disease: two randomised clinical trials Lancet 2009 374 685 694 10.1016/S0140-6736(09)61255-1 19716960

[B9] Fabbri LM Calverley PM Izquierdo-Alonso JL Bundschuh DS Brose M Martinez FJ Rabe KF M2-127 and M2-128 study groups Roflumilast in moderate-to-severe chronic obstructive pulmonary disease treated with longacting bronchodilators: two randomised clinical trials Lancet 2009 374 695 703 10.1016/S0140-6736(09)61252-6 19716961

[B10] Pryzwansky KB Madden VJ Type 4A cAMP-specific phosphodiesterase is stored in granules of human neutrophils and eosinophils Cell Tissue Res 2003 312 301 311 10.1007/s00441-003-0728-y 12764607

[B11] Peter D Jin SL Conti M Hatzelmann A Zitt C Differential expression and function of phosphodiesterase 4 (PDE4) subtypes in human primary CD4+ T cells: predominant role of PDE4 J Immunol 2007 178 4820 4831 17404263 10.4049/jimmunol.178.8.4820

[B12] Sawai T Ikai K Uehara M Cyclic adenosine monophosphate phosphodiesterase activity in peripheral blood mononuclear leucocytes from patients with atopic dermatitis: correlation with respiratory atopy Br J Dermatol 1998 138 846 848 10.1046/j.1365-2133.1998.02223.x 9666832

[B13] Hanifin JM Chan SC Monocyte phosphodiesterase abnormalities and dysregulation of lymphocyte function in atopic dermatitis J Invest Dermatol 1995 105 1 Suppl 84S 8S 7616004 10.1111/1523-1747.ep12316116

[B14] Hatzelmann A Morcillo EJ Lungarella G Adnot S Sanjar S Beume R Schudt C Tenor H The preclinical pharmacology of roflumilast--a selective, oral phosphodiesterase 4 inhibitor in development for chronic obstructive pulmonary disease Pulm Pharmacol Ther 2010 23 235 256 10.1016/j.pupt.2010.03.011 20381629

[B15] Boulet LP Gauvreau G Boulay ME O'Byrne P Cockcroft DW The allergen bronchoprovocation model: an important tool for the investigation of new asthma anti-inflammatory therapies Allergy 2007 62 1101 1110 10.1111/j.1398-9995.2007.01499.x 17845579

[B16] O'Byrne PM Dolovich J Hargreave FE Late asthmatic responses Am Rev Respir Dis 1987 136 740 751 10.1164/ajrccm/136.3.740 3115156

[B17] Gauvreau GM Lee JM Watson RM Irani AM Schwartz LB O'Byrne PM Increased numbers of both airway basophils and mast cells in sputum after allergen inhalation challenge of atopic asthmatics Am J Respir Crit Care Med 2000 161 1473 1478 10806141 10.1164/ajrccm.161.5.9908090

[B18] Gauvreau GM Watson RM O'Byrne PM Kinetics of allergen-induced airway eosinophilic cytokine production and airway inflammation Am J Respir Crit Care Med 1999 160 640 647 10430741 10.1164/ajrccm.160.2.9809130

[B19] Aalbers R Kauffman HF Vrugt B Koeter GH de Monchy JG Allergen-induced recruitment of inflammatory cells in lavage 3 and 24 h after challenge in allergic asthmatic lungs Chest 1993 103 1178 1184 10.1378/chest.103.4.1178 8131461

[B20] Woolley KL Adelroth E Woolley MJ Ellis R Jordana M O'Byrne PM Effects of allergen challenge on eosinophils, eosinophil cationic protein, and granulocyte-macrophage colony-stimulating factor in mild asthma Am J Respir Crit Care Med 1995 151 1915 1924 7767540 10.1164/ajrccm.151.6.7767540

[B21] Bentley AM Meng Q Robinson DS Hamid Q Kay AB Durham SR Increases in activated T lymphocytes, eosinophils, and cytokine mRNA expression for interleukin-5 and granulocyte/macrophage colony-stimulating factor in bronchial biopsies after allergen inhalation challenge in atopic asthmatics Am J Respir Cell Mol Biol 1993 8 35 42 8417755 10.1165/ajrcmb/8.1.35

[B22] Nouri-Aria KT Irani AM Jacobson MR O'brien F Varga EM Till SJ Durham SR Schwartz LB Basophil recruitment and IL-4 production during human allergen-induced late asthma J Allergy Clin Immunol 2001 108 205 211 10.1067/mai.2001.117175 11496235

[B23] Gauvreau GM Boulet LP Cote J Deschesnes F Duong M Killian K Obminski G Strinich TX Watson RM Cerasoli F Schmid-Wirlitsch C Bredenbröker D O'Byrne PM Effects of roflumilast on airway inflammation and function following allergen challenge in patients with allergic asthma [abstract] Am J Respir Crit Care Med 2007 175 A484

[B24] Bateman ED Izquierdo JL Harnest U Hofbauer P Magyar P Schmid-Wirlitsch C Leichtl S Bredenbröker D Efficacy and safety of roflumilast in the treatment of asthma Ann Allergy Asthma Immunol 2006 96 679 686 10.1016/S1081-1206(10)61065-4 16729780

[B25] Cockcroft DW Hargreave FE, Woolcock AJ Measure of airway responsiveness to inhaled histamine or methacholine; method of continuous aerosol generation and tidal breathing inhalation Airway responsiveness: measurement and interpretation 1985 Mississauga: Astra Pharmaceuticals Canada Ltd 22 28

[B26] Cockcroft DW Murdock KY Kirby J Hargreave F Prediction of airway responsiveness to allergen from skin sensitivity to allergen and airway responsiveness to histamine Am Rev Respir Dis 1987 135 264 267 3800152 10.1164/arrd.1987.135.1.264

[B27] Pizzichini E Pizzichini MM Efthimiadis A Evans S Morris MM Squillace D Gleich GJ Dolovich J Hargreave FE Indices of airway inflammation in induced sputum: reproducibility and validity of cell and fluid-phase measurements Am J Respir Crit Care Med 1996 154 308 317 8756799 10.1164/ajrccm.154.2.8756799

[B28] Bethke TD Giessmann T Westphal K Weinbrenner A Hauns B Hauschke D David M Lahu G Zech K Hermann R Siegmund W Roflumilast, a once-daily oral phosphodiesterase 4 inhibitor, lacks relevant pharmacokinetic interactions with inhaled salbutamol when co-administered in healthy subjects Int J Clin Pharmacol Ther 2006 44 572 579 17176624 10.5414/cpp44572

[B29] Harbinson PL MacLeod D Hawksworth R O'Toole S Sullivan PJ Heath P Kilfeather S Page CP Costello J Holgate ST Lee TH The effect of a novel orally active selective PDE4 isoenzyme inhibitor (CDP840) on allergen-induced responses in asthmatic subjects Eur Respir J 1997 10 1008 1014 10.1183/09031936.97.10051008 9163639

[B30] Louw C Williams Z Venter L Leichtl S Schmid-Wirlitsch C Bredenbroker D Bardin PG Roflumilast, a phosphodiesterase 4 inhibitor, reduces airway hyperresponsiveness after allergen challenge Respiration 2007 74 411 417 10.1159/000095677 16954654

[B31] van Schalkwyk E Strydom K Williams Z Venter L Leichtl S Schmid-Wirlitsch C Bredenbröker D Bardin PG Roflumilast, an oral, once-daily phosphodiesterase 4 inhibitor, attenuates allergen-induced asthmatic reactions J Allergy Clin Immunol 2005 116 292 298 10.1016/j.jaci.2005.04.023 16083782

[B32] Grootendorst DC Gauw SA Verhoosel RM Sterk PJ Hospers JJ Bredenbroker D Bethke TD Hiemstra PS Rabe KF Reduction in sputum neutrophil and eosinophil numbers by the PDE4 inhibitor roflumilast in patients with COPD Thorax 2007 62 1081 1087 10.1136/thx.2006.075937 17573446 PMC2094292

[B33] Sanz MJ Cortijo J Taha MA Cerda-Nicolas M Schatton E Burgbacher B Klar J Tenor H Schudt C Issekutz AC Hatzelmann A Morcillo EJ Roflumilast inhibits leukocyte-endothelial cell interactions, expression of adhesion molecules and microvascular permeability Br J Pharmacol 2007 152 481 492 10.1038/sj.bjp.0707428 17704822 PMC2050829

[B34] Hatzelmann A Schudt C Anti-inflammatory and immunomodulatory potential of the novel PDE4 inhibitor roflumilast in vitro J Pharmacol Exp Ther 2001 297 267 279 11259554

[B35] Gauvreau GM Doctor J Watron R Jordana M O'Byrne PM Effects of inhaled budesonide on allergen-induced airway responses and airway inflammation Am J Respir Crit Care Med 1996 154 1267 1271 8912734 10.1164/ajrccm.154.5.8912734

[B36] Cowan DC Cowan JO Palmay R Williamson A Taylor DR Effects of steroid therapy on inflammatory cell subtypes in asthma Thorax 2010 65 384 390 10.1136/thx.2009.126722 19996343

[B37] Grootendorst DC Gauw SA Baan R Kelly J Murdoch RD Sterk PJ Rabe KF Does a single dose of the phosphodiesterase 4 inhibitor, cilomilast (15 mg), induce bronchodilation in patients with chronic obstructive pulmonary disease? Pulm Pharmacol Ther 2003 16 115 120 10.1016/S1094-5539(02)00172-4 12670781

